# The causal role of C-reactive protein and interleukin-6 on anxiety and depression symptoms and life satisfaction: Mendelian randomisation analyses in the HUNT study

**DOI:** 10.1017/S0033291723001290

**Published:** 2023-12

**Authors:** Ole-Jørgen Bekkevold, Jan Kristian Damås, Ben Michael Brumpton, Bjørn Olav Åsvold

**Affiliations:** 1 K.G. Jebsen Center for Genetic Epidemiology, Department of Public Health and Nursing, NTNU, Norwegian University of Science and Technology, Trondheim, Norway; 2 Department of Infectious Diseases, Clinic of Medicine, St. Olavs Hospital, Trondheim University Hospital, Trondheim, Norway; 3 Department of Clinical and Molecular Medicine, Centre of Molecular Inflammation Research, NTNU, Norwegian University of Science and Technology, Trondheim, Norway; 4 HUNT Research Centre, Department of Public Health and Nursing, NTNU, Norwegian University of Science and Technology, Levanger, Norway; 5 Clinic of Medicine, St. Olavs Hospital, Trondheim University Hospital, Trondheim, Norway; 6 Department of Endocrinology, Clinic of Medicine, St. Olavs Hospital, Trondheim University Hospital, Trondheim, Norway

**Keywords:** Anxiety, C-reactive protein, depression, hospital anxiety and depression scale, interleukin-6, life satisfaction, Mendelian randomisation, the HUNT study

## Abstract

**Background:**

Serum levels of C-reactive protein (CRP) and interleukin-6 (IL-6) have been associated with anxiety and depression in cross-sectional and Mendelian randomisation studies, but results regarding the effect size and direction have been mixed. A recent Mendelian Randomisation (MR) study suggested that CRP may decrease and IL-6 may increase anxiety and depression symptoms.

**Methods:**

Among 68 769 participants of the population-based Trøndelag Health Study (HUNT), we performed cross-sectional observational and one-sample MR analyses of serum CRP and two-sample MR analysis of serum IL-6. The main outcomes were symptoms of anxiety and depression assessed using the Hospital Anxiety and Depression Scale (HADS) and life satisfaction assessed using a seven-level ordinal questionnaire where higher scores indicate lower life satisfaction.

**Results:**

In cross-sectional observational analyses, a doubling in serum CRP level was associated with 0.27% (95% CI −0.20 to 0.75) difference in HADS depression score (HADS-D), −0.77% (95% CI −1.24 to −0.29) difference in HADS anxiety score (HADS-A) and −0.10% (95% CI −0.41 to 0.21) difference in life satisfaction score. In one-sample MR analyses, a doubling in serum CRP was associated with 2.43% (95% CI −0.11 to 5.03) higher HADS-D, 1.94% (95% CI −0.58 to 4.52) higher HADS-A, and 2.00% (95% CI 0.45 to 3.59) higher life satisfaction score. For IL-6, causal point estimates were in the opposite direction, but imprecise and far from conventional criteria for statistical significance.

**Conclusions:**

Our results do not support a major causal role of serum CRP on anxiety and depression symptoms and life satisfaction, but provides weak evidence that serum CRP may modestly increase anxiety and depression symptoms and reduce life satisfaction. Our findings do not support the recent suggestion that serum CRP may lower anxiety and depression symptoms.

## Introduction

Depression and anxiety are common contributors to population ill health. Clinical depression and anxiety disorders are responsible for an estimated 8.8% of the total years lived with disability globally (Global Burden of Disease Collaborative Network, 2019). Prevention of depression and anxiety requires sound knowledge about their aetiology. Elevated serum levels of C-reactive protein (CRP) and the biologically linked interleukin-6 (IL-6), as seen in low-grade inflammation, are among the many proposed causes, and observational studies have reported higher serum CRP levels among people with depression (Gimeno et al., 2009; Howren, Lamkin, & Suls, 2009; Miller & Raison, 2016; Osimo, Baxter, Lewis, Jones, & Khandaker, 2019) and anxiety (Michopoulos, Powers, Gillespie, Ressler, & Jovanovic, 2017; Pitsavos et al., 2006; Vogelzangs, Beekman, de Jonge, & Penninx, 2013). However, it is uncertain whether these associations reflect causal effects of serum CRP, causality in the reverse direction (i.e. depression and anxiety causing increased serum CRP levels), or confounding due to common causes of elevated serum CRP and anxiety and depression.

Mendelian Randomisation (MR) may resolve the causal nature of these associations, by using genetically determined variation in serum CRP and serum IL-6 levels as instrumental variables to inform about their causal effects, while avoiding confounding and reverse causation bias (Haycock et al., 2016). However, MR studies have yielded inconsistent evidence, suggesting that higher genetically predicted serum CRP levels may either increase (Khandaker et al., 2020) or have no effect (Kim Wium-Andersen, Dynnes Ørsted, & Grønne Nordestgaard, 2014) on the risk of depression. In apparent contrast to those results, a recent MR study in the UK Biobank population by Ye et al., yielded evidence that serum CRP may reduce, whereas serum IL-6 may increase anxiety and depression symptoms (Ye et al., 2021). However, UK Biobank has a participation rate of only 5.5% (Fry et al., 2017), and is therefore prone to collider bias (Munafò, Tilling, Taylor, Evans, & Davey Smith, 2017). We aimed to investigate the effects of serum CRP and serum IL-6 on anxiety and depression symptoms and life satisfaction by using MR analyses in a study population less prone to collider bias, the population-based Trøndelag Health Study (HUNT) in Norway with 54% (HUNT3, 2006–08) to 69% (HUNT2, 1995–97) participation rates (Krokstad et al., 2013).

## Method

### Study design and study population

Using data from the HUNT Study, we estimated the cross-sectional associations of serum CRP with symptoms of depression and anxiety and life satisfaction and performed one-sample MR analyses to estimate the causal effects of serum CRP levels on these mental health outcomes. We used a two-sample MR approach to examine the corresponding causal effects of serum IL-6. For details about the HUNT study and its variables, see supplementary data, section ‘1. The HUNT study and variables’.

For our main analyses, we used similar genetic instruments for serum CRP as applied by Hartwig et al., in 2017 (Hartwig, Borges, Horta, Bowden, & Davey Smith, 2017). This included two instruments: ‘CRP-conservative’ contains single nucleotide polymorphisms (SNPs) in the *CRP* gene that showed strong associations with serum CRP levels in a meta-analysis of 194 418 individuals (Wensley et al., 2011). ‘CRP-liberal’ includes all other SNPs that were genome-wide significantly associated with serum CRP levels in a meta-analysis of 80 000 participants of European descent (Dehghan et al., 2011). We included the ‘CRP-conservative’ SNPs in our ‘CRP-liberal’ instrument. We put most emphasis on the results using the conservative instrument, as the restriction to the *CRP* gene makes horizontal pleiotropy less likely. The advantage of the liberal instrument is that the higher number of variants across the genome may increase statistical power, though at the cost of higher possibility of horizontal pleiotropy. As instruments for serum IL-6 levels we selected 3 SNPs within 55 kb of the *IL6R* gene that have been used in a recent MR study (Ye et al., 2021), originating from a 2012 genetic association study (Swerdlow et al., 2012) where HUNT was not a part of the meta-analysis.

As part of the sensitivity analyses, we conducted one-sample MR with an alternative CRP-instrument (denoted as ‘CRP-sensitivity’). This instrument has recently been used in several MR studies (Georgakis et al., 2020; Kappelmann et al., 2021; Ye et al., 2021) and contains 24 SNPs in the CRP region, based on a GWAS by Ligthart et al. (Ligthart et al. 2018). For more details about SNPs and genetic instruments, see supplementary data, section ‘2. Genetic instruments’.

Among 78 962 participants in HUNT2 or HUNT3, we excluded 9751 participants without genotype information and 442 participants with no information on either serum CRP, Hospital Anxiety and Depression Scale (HADS) or life satisfaction, leaving 68 769 participants of European decent for the MR-analysis. However, since we calculated SNP-exposure and SNP-outcome associations separately the population size differs for each phenotype according to how many missing values there were for that particular phenotype, see the ‘n exposure’ and ‘n outcome’ column in online Supplementary Figs S3–S6. In the cross-sectional analysis, we required that all variables and covariates for each individual were collected at the same time point. Therefore we additionally excluded 29 935 participants that did not have data on serum CRP, HADS, life satisfaction, BMI, diabetes, alcohol use, cardiovascular disease and smoking status from the same survey (HUNT2 or HUNT3), leaving 38 834 participants for analysis.

### Statistical methods

HADS and life satisfaction scores were natural log transformed due to skewed distributions, and serum CRP was log transformed with base 2 so that all outcomes are reported per doubling in CRP. We added 0.1 to all serum CRP values and 1 to all HADS values before transformation to avoid log(0) values. For participants with data from both HUNT2 and HUNT3 we used the earliest measurement, as these measurements were likely less influenced by confounding factors occurring more commonly in older age.

To estimate the cross-sectional associations of the measured serum CRP levels with HADS and life satisfaction, we used multivariable linear regression to estimate the percentage difference in HADS and life satisfaction scores per doubling in serum CRP, and used logistic regression to estimate the odds ratio (OR) of elevated HADS-A or HADS-D per doubling in serum CRP. We adjusted for age, sex, BMI, alcohol use, cardiovascular disease, smoking status and diabetes.

For the one-sample MR analysis we estimated the SNP-CRP and SNP-outcome associations using individual-level HUNT information on genotypes, serum CRP and HADS/life satisfaction. To account for relatedness in the HUNT population, we used a linear mixed effects model, with BOLT-LMM software (Loh et al., 2015), to control for sex, birth year, genotyping batch and the first 4 genetic principal components. The output from BOLT-LMM represents the linear relationship between dosage of the individual SNP and the phenotype. Since the outcome-scores were log transformed, we converted the beta estimate to percentage change by the following formula:
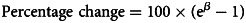


For the binary phenotypes we used the following formula from the BOLM-LMM User manual to approximate the log (OR):
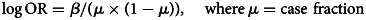


The standard errors for the log (OR) were approximated by the following formula:
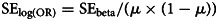


For the one-sample MR we used the SNP-exposure and SNP-outcome associations from the BOLT-LMM software and calculated inverse variance weighted (IVW) MR estimates using the ‘TwoSampleMR’ package (Hemani et al., 2018) version 0.5.6, combined with the ‘MendelianRandomisation’ package (Olena Yavorska, 2021) version 0.5.1, which allowed us to account for linkage disequilibrium between the SNPs.

For the two-sample MR analyses we used SNP-IL-6 associations as reported in Ye et al. (Ye et al. 2021), and SNP-outcome associations from the BOLT-LMM analysis using individual genotype and outcome information from HUNT. In the same manner as for the one-sample MR we combined the ‘TwoSampleMR’ package (Hemani et al., 2018) and the ‘MendelianRandomisation’ package (Olena Yavorska, 2021) to obtain IVW MR estimates accounting for linkage disequilibrium.

Except for the mixed effect model analysis, all statistical analyses were performed in R (R Core Team, 2021) version 4.0.5. Mixed effect model analysis was performed with the BOLT-LMM software (Loh et al., 2015) (version 2.3.4).

### Assessment of MR assumptions and other sensitivity analyses

MR builds on three main assumptions: (1) the relevance assumption that the genetic instrument is associated with the exposure; (2) the exclusion restriction assumption that there should be no other mechanism in which the SNP affects the outcome other than through the exposure (a violation of which would cause horizontal pleiotropy), and (3) the independence assumption that the SNP should be independent from any confounder of the exposure-outcome association. To evaluate the plausibility of these assumptions we used several different methods including calculation of *F*-statistics and *R*^2^, Phenoscanner, regression analyses between SNPs and possible confounders, Cochran *Q*-statistic, MR Egger, median weighted MR, weighted mode MR and MR with an alternative genetic instrument. For details on how this was conducted, see supplementary data, section ‘3. Method for assessment of MR assumptions and other sensitivity analyses’.

## Results

### Descriptive data

The characteristics of the study population are described in Table 1.

**Table 1.** Characteristics of study populations

Characteristics	MR population (*n* = 68 769)	Observational population (*n* = 38 834)
Birthyear, median (IQR)	1951 (1937–1965)	1953 (1942–1964)
Sex, % males	47.0	45.3
BMI (kg/m^^2^), mean (s.d.)	26.4 (4.2)	27.0 (4.3)
Diabetes, %	3.0	4.0
History of stroke, %	1.8	2.3
History of myocardial infarction, %	2.7	3.0
Current smokers, %	29.0	24.4
Total alcohol units per week, median (IQR)	1 (0–3)	2 (1–3)
CRP, median (IQR) mg/L	1.2 (0.6–2.7)	1.2 (0.5–2.6)
HADS-anxiety score ≥ 8, %	15.5	14.4
HADS-anxiety score, median (IQR)	4 (2–6)	3 (2–6)
HADS-depression score ≥ 8, %	10.4	9.4
HADS-depression score, median (IQR)	3 (1–5)	3 (1–5)
Total HADS score, median (IQR)	6.5 (3.2–10.8)	6 (3–10)
Life satisfaction score, median (IQR)	3 (2–3)	2 (2–3)
Missing information on CRP, n	17 423	0
Missing information on HADS-A, n	5394	0
Missing information on HADS-D, n	4716	0
Missing information on total HADS, n	5408	0
Missing information on life satisfaction, n	417	0

IQR, interquartile range; s.d., standard deviation; n, number.

### Cross-sectional analysis

**Figure 1.**
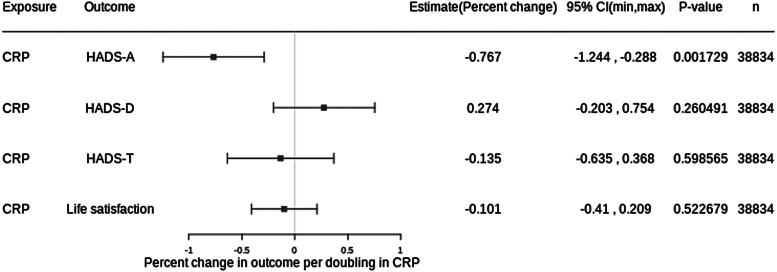
Results from cross-sectional analysis with continuous outcomes. Adjusted for age, sex, BMI, alcohol use, cardiovascular disease, smoking status and diabetes. CRP, C-reactive protein; HADS, hospital anxiety and depression scale; HADS-A, HADS-anxiety score; HADS-D, HADS-depression score; HADS-T, total HADS score; 95% CI, 95% confidence interval; n, number of participants.

**Figure 2.**
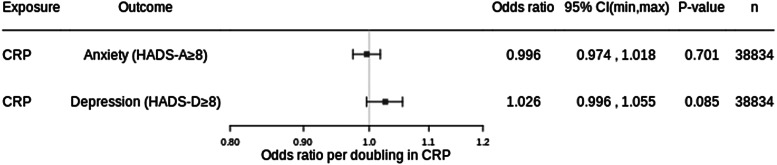
Results from cross-sectional analysis with binary outcomes. Adjusted for age, sex, BMI, alcohol use, cardiovascular disease, smoking status and diabetes. CRP, C-reactive protein; HADS, hospital anxiety and depression scale; HADS-A, HADS-anxiety score; HADS-D, HADS-depression score; HADS-T, total HADS score; 95% CI, 95% confidence interval; n, number of participants.

Adjusted for age, sex, BMI, alcohol use, cardiovascular disease, smoking status and diabetes, each doubling in serum CRP was associated with −0.14% difference in HADS-T (95% CI −0.64 to 0.37, *p* value: 0.599), 0.27% difference in HADS-D (95% CI −0.20 to 0.75; *p* value: 0.260) and −0.77% difference in HADS-A (95% CI −1.24 to −0.29; *p* value: 0.002) (Fig. 1). For binary outcomes, each doubling in serum CRP was associated with an OR of 1.03 (95% CI 0.996 to 1.06; *p* value: 0.085) for HADS-D ≥ 8 and an OR of 0.996 (95% CI 0.97 to 1.02, *p* value: 0.701) for HADS-A ≥ 8 (Fig. 2). There was a difference of −0.10% in life satisfaction score per doubling in CRP (95% CI −0.41 to 0.21; *p* value: 0.523) (Fig. 1).

### One-sample MR analysis of CRP serum levels

**Figure 3.**
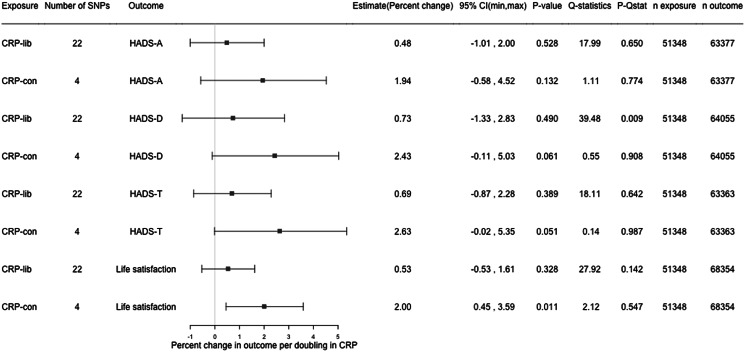
Results from one-sample MR with continuous outcomes. CRP, C-reactive protein; CRP-lib, CRP-liberal genetic instrument; CRP-con, CRP-conservative genetic instrument; SNP, single nucleotide polymorphism; HADS, hospital anxiety and depression scale; HADS-A, HADS-anxiety score; HADS-D, HADS-depression score; HADS-T, total HADS score; 95% CI, 95% confidence interval; *p*-Qstat, *p* value for *Q*-statistic; n exposure, number of participants in SNP-exposure analysis; n outcome, number of participants in SNP-outcome analysis.

**Figure 4.**
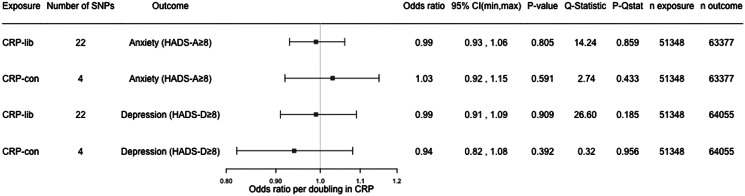
Results from one-sample MR with binary outcomes. CRP, C-reactive protein; CRP-lib, CRP-liberal genetic instrument; CRP-con, CRP-conservative genetic instrument; SNP, single nucleotide polymorphism; HADS, hospital anxiety and depression scale; HADS-A, HADS-anxiety score; HADS-D, HADS-depression score; 95% CI, 95% confidence interval; *p*-Qstat, *p* value for *Q*-statistic; n exposure, number of participants in SNP-exposure analysis; n outcome, number of participants in SNP-outcome analysis.

In one-sample MR analysis using the conservative genetic instrument for CRP, each doubling in genetically predicted serum CRP was associated with 2.63% (95% CI −0.02 to 5.35, *p* value: 0.051) higher HADS-T score, 2.43% (95% CI −0.11 to 5.03, *p* value: 0.061) higher HADS-D score, 1.94% (95% CI −0.58 to 4.52, *p* value: 0.132) higher HADS-A score, and 2.00% (95% CI 0.45–3.59, *p* value: 0.011) higher life satisfaction score. The associations were in the same direction, but weaker for the liberal instrument (Fig. 3). A doubling in genetically predicted serum CRP gave an OR of 0.94 (95% CI 0.82–1.08, *p* value: 0.392) for HADS-D ≥ 8, and 1.03 (95% CI 0.92–1.15, *p* value: 0.591) for HADS-A ≥ 8 (Fig. 4).

### Two-sample MR analysis of IL-6 serum levels

**Figure 5.**
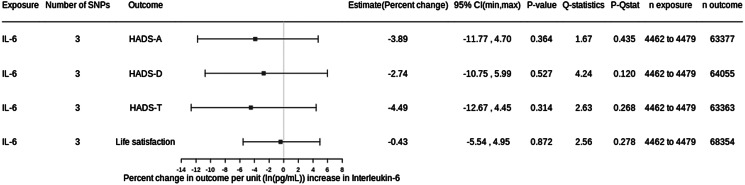
Results from two-sample MR with continuous outcomes. CRP, C-reactive protein; IL-6, interleukin-6; SNP, single nucleotide polymorphism; HADS, hospital anxiety and depression scale; HADS-A, HADS-anxiety score; HADS-D, HADS-depression score; HADS-T, total HADS score; 95% CI, 95% confidence interval; *p*-Qstat, *p* value for *Q*-statistic; n exposure, number of participants in SNP-exposure analysis; n outcome, number of participants in SNP-outcome analysis.

**Figure 6.**
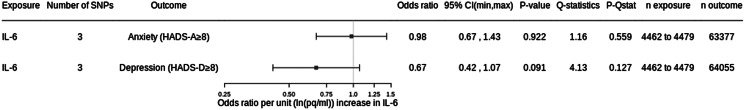
Results from two-sample MR with binary outcomes. CRP, C-reactive protein; IL-6, interleukin-6; SNP, single nucleotide polymorphism; HADS, hospital anxiety and depression scale; HADS-A, HADS anxiety score; HADS-D, HADS depression score; 95% CI, 95% confidence interval; *p*-Qstat, *p* value for *Q*-statistic; n exposure, number of participants in SNP-exposure analysis; n outcome, number of participants in SNP-outcome analysis.

Genetically predicted higher serum IL-6 were associated with slightly lower HADS-T, HADS-D, HADS-A and life satisfaction scores, but all estimates were hampered by large imprecision (Fig. 5). For clinical depression and anxiety, signs of an inverse association were only seen for depression (Fig. 6); however, no association with IL-6 reached conventional criteria for statistical significance.

### Assessment of assumptions

#### Relevance assumption

*F*-statistics for the SNP-exposure associations were >214 for all SNPs included in the conservative CRP instrument, >10 for 14 of the 18 SNPs additionally included in the liberal CRP instrument, and >22 for all SNPs used in the IL-6 instrument (online Supplementary Tables S5–S7). This indicates that 4 of the SNPs in the liberal instrument were too weak, which in a one-sample setting would bias the MR estimate towards the cross-sectional estimate (Davies, Holmes, & Davey Smith, 2018).

To investigate this further, we ran linear regression with the allele count for the conservative and the liberal CRP-instrument. This yielded an adjusted *R*^2^ of 0.019 for the conservative and 0.038 for the liberal instrument. The *F*-statistics were 989.9 and 2025.2 respectively (online Supplementary Table S8). This indicates that even if some SNPs may be too weak, the general strength of the liberal instrument is satisfactory and the risk for weak instrument bias is small.

#### Exclusion restriction assumption and independence assumption

Using the R-package Phenoscanner (Kamat et al., 2019; Staley et al., 2016) to search through the NHGRI-EBI GWAS-catalog (Buniello et al., 2019), we found no genome-wide significant associations between the four SNPs in the conservative CRP instrument and other phenotypes than CRP (online Supplementary Table S9). However, many of the 18 additional SNPs in the liberal CRP instrument were associated with traits that could be potential confounders or pleiotropic factors, in particular CVD and CVD risk factors (online Supplementary Table S10). Among SNPs used in the instrument for IL-6, we found a few associations with potential confounders such as CVD, eczema and rheumatoid arthritis (online Supplementary Table S11). These associations may reflect that IL-6 may increase the risk of these diseases, which would not imply a violation of the independence assumption. Analyses within the HUNT study population showed no strong associations between the allele scores for the genetic instruments for CRP and the potential pleiotropic factors such as BMI, smoking status, or a history of myocardial infarction, heart failure or rheumatoid arthritis (online Supplementary Tables S12 and S13). For the IL-6 instrument there were no statistically significant associations between allele score and likely confounders or pleiotropic factors (online Supplementary Table S14).

The MR-Egger, weighted median MR and weighted mode MR analyses were quite consistent with the IVW analysis in that they did not point to large effects of serum CRP on the outcomes (online Supplementary Figs S1 and S2). The MR Egger test (pleiotropy *p* value) did not indicate any large directional pleiotropy. The *Q*-statistics for the conservative and liberal CRP instruments (online Supplementary Figs S3 and S4) indicated no strong heterogeneity, except for the liberal CRP instrument with HADS-D as outcome, with a *Q*-statistic of 39.48 (*p* value 0.009) suggesting a violation of the exclusion restriction assumption. Since there only were three SNPs in the IL-6 instrument, we did not run MR-Egger, weighted median or weighted mode MR for IL-6. The *Q*-statistics for the IL-6 SNPs were relatively small, all below 5 with *p* values ≥ 0.120, suggesting little heterogeneity (Figs 5 and 6).

### Sensitivity analysis

The results from IVW MR using the alternative genetic instrument from Ligthart et al. (Ligthart et al. 2018) combined with the CRP-conservative instrument were consistent with the main results (online Supplementary Figs S3 and S4). The *Q*-statistics for this instrument showed no strong signs of heterogeneity and MR-Egger did not indicate any problems with pleiotropy with all pleiotropy *p* values above 0.05 (online Supplementary Figs S5 and S6). However, with *F*-statistics below 10 for 5 of the 26 SNPs and an overall *F*-statistic for the entire instrument of 723, this instrument was weaker than the CRP liberal instrument in HUNT (online Supplementary Tables S15 and S16).

## Discussion

### Key results

We observed weak cross-sectional associations where each doubling in serum CRP was associated with approximately 0.3% higher HADS-D score and −0.1% difference in life satisfaction score (where lower scores indicate higher life satisfaction), but −0.8% difference in HADS-A score. Only the association with HADS-A reached conventional criteria for statistical significance. MR analyses provided some evidence that serum CRP may modestly increase anxiety and depression symptoms and reduce life satisfaction, with ~2% higher scores per doubling in serum CRP, but only the association with life satisfaction reached conventional criteria for statistical significance. For IL-6, causal estimates were in the opposite direction, but imprecise and far from conventional criteria for statistical significance.

### Limitations

Our sensitivity analyses indicate that the core MR assumptions are held for the conservative CRP instrument, whereas associations to possible confounders and signs of heterogeneity suggest that the estimates for the liberal CRP instrument should be interpreted with more caution. Applying a different set of SNPs from a recent GWAS (Ligthart et al., 2018), the CRP-sensitivity instrument revealed results consistent with the liberal CRP instrument. This instrument showed no signs of heterogeneity or pleiotropy, but had weaker *F*-statistics and *R*^2^ than the CRP liberal instrument. Weak instrument bias could potentially bias the estimates for the CRP liberal and CRP sensitivity instruments towards the cross-sectional estimate (Davies et al., 2018). Future studies could use a two-sample design to avoid this, but also to gain more statistical power to precisely estimate weak causal estimates like those we observed. Another limitation of this study is that the measurement of life satisfaction only included a single question, making it difficult to evaluate what aspects of life satisfaction were captured.

### Interpretations

CRP has been suggested to influence mental health in previous studies. Even though CRP does not pass the blood brain barrier freely, CRP could affect the central nervous system via passage through leaky regions, active uptake, activation of endothelial cells and immunity cells or binding to receptors on peripheral nerves (Felger et al., 2020). Our findings, however, do not support a major causal role of serum CRP as a determinant of anxiety and depression symptoms and life satisfaction. Nonetheless, our MR analyses provide weak evidence that serum CRP may modestly increase anxiety and depression symptoms and lower life satisfaction, which would be in agreement with conventional observational studies showing serum CRP to be elevated in a quarter of patience with depression (Osimo et al., 2019) and serum CRP to be elevated in subgroups of patients with anxiety disorders (Vogelzangs et al., 2013). Also, such a causal effect would be in agreement with the hypothesis that inflammation may increase sickness behaviour and fatigue which again would affect mental health (Miller & Raison, 2016).

Among MR studies, Khandaker et al. (Khandaker et al. 2020) found increased risk of lifetime major depression at higher genetically predicted levels of serum CRP and IL-6 in UK Biobank. In contrast, Kappelman et al. (Kappelmann et al. 2021) and Wium Andersen et al. (Kim Wium-Andersen et al. 2014) found no effect of serum CRP on depression, whereas Perry et al. (Perry et al. 2021) found no effect of serum CRP, but evidence of an effect of higher serum IL-6 on depression. In opposite direction of our findings, evidence from the recent UK Biobank MR study by Ye et al. (Ye et al. 2021) suggested that serum CRP may lower, while serum IL-6 may increase anxiety and depression symptoms. The reasons for the discrepancy between our results and those of Ye et al., are not known, but the low participation rate in UK Biobank could give rise to collider bias (Munafò et al., 2017). Ye et al., comprehensively evaluated the risk for potential collider bias with inverse probability weighting techniques and found no evidence of bias. However, the ability of such analyses to detect and correct for selection bias depends on how accurately the probability of selection can be modelled (Gkatzionis & Burgess, 2019). We also note that Ye et al., assessed symptoms using the Patient Health Questionnaire (PHQ-9) and the Generalised Anxiety Disorder (GAD-7) Questionnaire, whereas we used HADS-D and HADS-A. PHQ-9 assesses 9 symptoms from the Diagnostic and Statistical Manual of Mental Disorders (DSM-IV) criteria for depression, while GAD-7 asseses 7 symptoms related to general anxiety disorder based on the DSM-IV criteria (Spitzer, Kroenke, Williams, & Löwe, 2006). One important difference between these questionaires and HADS, is that HADS does not meassure somatic symptoms of depression and anxiety such as dizziness, headaches, insomnia and fatigue. HADS also excludes symptoms related to serious mental disorders (Bjelland, Dahl, Haug, & Neckelmann, 2002). However, studies show that HADS-D and HADS-A are comparable with PHQ-9 and GAD-7 in their psychometric properties (Esser et al., 2018; Hansson, Chotai, Nordstöm, & Bodlund, 2009). Hence, comparing our results from HADS with PHQ-9 and GAD-7 is sensible.

### Generalisability

Our study is based on a Norwegian population from Trøndelag County with low ethnic diversity. However, it seems unlikely that serum CRP and serum IL-6 should have major different effects on anxiety and depression symptoms in this group compared with other populations.

### Conclusion

In conclusion our findings do not support a major causal role of serum CRP on anxiety and depression symptoms and life satisfaction, but provide weak evidence that serum CRP may modestly decrease life satisfaction and increase anxiety and depression symptoms. For IL-6, causal estimates were in the opposite direction, but impresise and therefore inconclusive. Our results do not support the recent suggestion that serum CRP may lower anxiety and depression symptoms.

## Supporting information

Bekkevold et al. supplementary material Bekkevold et al. supplementary material

## Supplementary material

The supplementary material for this article can be found at https://doi.org/10.1017/S0033291723001290.

## References

[ref1] Bjelland, I., Dahl, A. A., Haug, T. T., & Neckelmann, D. (2002). The validity of the Hospital Anxiety and Depression Scale: An updated literature review. Journal of Psychosomatic Research, 52, 69–77. doi:10.1016/S0022-3999(01)00296-3. 11832252

[ref2] Buniello, A., MacArthur, J. A. L., Cerezo, M., Harris, L. W., Hayhurst, J., Malangone, C., … Parkinson, H. (2019). The NHGRI-EBI GWAS Catalog of published genome-wide association studies, targeted arrays and summary statistics 2019. Nucleic Acids Research, 47(D1), D1005–D1012. doi:10.1093/nar/gky1120. 30445434 PMC6323933

[ref3] Davies, N. M., Holmes, M. V., & Davey Smith, G. (2018). Reading Mendelian randomisation studies: A guide, glossary, and checklist for clinicians. British Medical Journal, 362, k601. doi:10.1136/bmj.k601. 30002074 PMC6041728

[ref4] Dehghan, A., Dupuis, J., Barbalic, M., Bis, J. C., Eiriksdottir, G., Lu, C., … Chasman, D. I. (2011). Meta-analysis of genome-wide association studies in >80 000 subjects identifies multiple loci for C-reactive protein levels. Circulation, 123, 731–738. doi:10.1161/CIRCULATIONAHA.110.948570. 21300955 PMC3147232

[ref5] Esser, P., Hartung, T. J., Friedrich, M., Johansen, C., Wittchen, H.-U., Faller, H., … Mehnert, A. (2018). The generalized anxiety disorder screener (GAD-7) and the anxiety module of the hospital and depression scale (HADS-A) as screening tools for generalized anxiety disorder among cancer patients. Psycho-Oncology, 27(6), 1509–1516. doi:10.1002/pon.4681. 29473255

[ref6] Felger, J. C., Haroon, E., Patel, T. A., Goldsmith, D. R., Wommack, E. C., Woolwine, B. J., … Miller, A. H. (2020). What does plasma CRP tell us about peripheral and central inflammation in depression? Molecular Psychiatry, 25(6), 1301–1311. doi:10.1038/s41380-018-0096-3. 29895893 PMC6291384

[ref7] Fry, A., Littlejohns, T. J., Sudlow, C., Doherty, N., Adamska, L., Sprosen, T., … Allen, N. E. (2017). Comparison of sociodemographic and health-related characteristics of UK Biobank participants with those of the general population. American Journal of Epidemiology, 186(9), 1026–1034. doi:10.1093/aje/kwx246. 28641372 PMC5860371

[ref8] Georgakis, M. K., Malik, R., Gill, D., Franceschini, N., Sudlow, C. L. M., Dichgans, M., … Slagboom, E. P. (2020). Interleukin-6 signaling effects on ischemic stroke and other cardiovascular outcomes. Circulation: Genomic and Precision Medicine, 13(3), e002872. doi:10.1161/CIRCGEN.119.002872. 32397738 PMC7299212

[ref9] Gimeno, D., Kivimäki, M., Brunner, E. J., Elovainio, M., De Vogli, R., Steptoe, A., … Ferrie, J. E. (2009). Associations of C-reactive protein and interleukin-6 with cognitive symptoms of depression: 12-year follow-up of the Whitehall II study. Psychological Medicine, 39, 413–423. doi:10.1017/S0033291708003723. 18533059 PMC2788760

[ref10] Gkatzionis, A., & Burgess, S. (2019). Contextualizing selection bias in Mendelian randomization: How bad is it likely to be? International Journal of Epidemiology, 48(3), 691–701. doi:10.1093/ije/dyy202. 30325422 PMC6659463

[ref11] Global Burden of Disease Collaborative Network. (2019). Global Burden of Disease Study 2019 (GBD 2019) Results. Retrieved from https://vizhub.healthdata.org/gbd-results/.

[ref12] Hansson, M., Chotai, J., Nordstöm, A., & Bodlund, O. (2009). Comparison of two self-rating scales to detect depression: HADS and PHQ-9. British Journal of General Practice, 59(566), e283–e288. doi:10.3399/bjgp09X454070. PMC2734374 19761655

[ref13] Hartwig, F. P., Borges, M. C., Horta, B. L., Bowden, J., & Davey Smith, G. (2017). Inflammatory biomarkers and risk of schizophrenia. JAMA Psychiatry, 74, 1226. doi:10.1001/jamapsychiatry.2017.3191. 29094161 PMC6583386

[ref14] Haycock, P. C., Burgess, S., Wade, K. H., Bowden, J., Relton, C., & Smith, G. D. (2016). Statistical commentary best (but oft-forgotten) practices : The design, analysis, and interpretation of Mendelian randomization studies 1. The American Journal of Clinical Nutrition, 103, 965–978. doi:10.3945/ajcn.115.118216.INTRODUCTION. 26961927 PMC4807699

[ref15] Hemani, G., Zheng, J., Elsworth, B., Wade, K. H., Haberland, V., Baird, D., … Haycock, P. C. (2018). The MR-Base platform supports systematic causal inference across the human phenome. eLife, 7, e34408. doi:10.7554/eLife.34408. 29846171 PMC5976434

[ref16] Howren, M. B., Lamkin, D. M., & Suls, J. (2009). Associations of depression with C-reactive protein, IL-1, and IL-6: A meta-analysis. Psychosomatic Medicine, 71(2), 171–186. doi:10.1097/PSY.0b013e3181907c1b. 19188531

[ref17] Kamat, M. A., Blackshaw, J. A., Young, R., Surendran, P., Burgess, S., Danesh, J., … Staley, J. R. (2019). PhenoScanner V2: An expanded tool for searching human genotype-phenotype associations. Bioinformatics (Oxford, England), 35(22), 4851–4853. doi:10.1093/bioinformatics/btz469. 31233103 PMC6853652

[ref18] Kappelmann, N., Arloth, J., Georgakis, M. K., Czamara, D., Rost, N., Ligthart, S., … Binder, E. B. (2021). Dissecting the association between inflammation, metabolic dysregulation, and specific depressive symptoms: A genetic correlation and 2–sample Mendelian randomization study. JAMA Psychiatry, 78(2), 161–170. doi:10.1001/jamapsychiatry.2020.3436. 33079133 PMC7577200

[ref19] Khandaker, G. M., Zuber, V., Rees, J. M. B., Carvalho, L., Mason, A. M., Foley, C. N., … Burgess, S. (2020). Shared mechanisms between coronary heart disease and depression: Findings from a large UK general population-based cohort. Molecular Psychiatry, 25(7), 1477–1486. doi:10.1038/s41380-019-0395-3. 30886334 PMC7303009

[ref20] Kim Wium-Andersen, M., Dynnes Ørsted, D., & Grønne Nordestgaard, B. (2014). Elevated C-reactive protein, depression, somatic diseases, and all-cause mortality: A Mendelian randomization study. Biological Psychiatry, 76, 249–257. doi:10.1016/j.biopsych.2013.10.009. 24246360

[ref21] Krokstad, S., Langhammer, A., Hveem, K., Holmen, T. L., Midthjell, K., Stene, T. R., … Holmen, J. (2013). Cohort profile: The HUNT study, Norway. International Journal of Epidemiology, 42(4), 968–977. doi:10.1093/ije/dys095. 22879362

[ref22] Ligthart, S., Vaez, A., Võsa, U., Stathopoulou, M. G., de Vries, P. S., Prins, B. P., … Alizadeh, B. Z. (2018). Genome analyses of >200,000 individuals identify 58 loci for chronic inflammation and highlight pathways that link inflammation and complex disorders. American Journal of Human Genetics, 103, 691–706. doi:10.1016/j.ajhg.2018.09.009. 30388399 PMC6218410

[ref23] Loh, P.-R., Tucker, G., Bulik-Sullivan, B. K., Vilhjálmsson, B. J., Finucane, H. K., Salem, R. M., … Price, A. L. (2015). Efficient Bayesian mixed-model analysis increases association power in large cohorts. Nature Genetics, 47(3), 284–290. doi:10.1038/ng.3190. 25642633 PMC4342297

[ref24] Michopoulos, V., Powers, A., Gillespie, C. F., Ressler, K. J., & Jovanovic, T. (2017). Inflammation in fear- and anxiety-based disorders: PTSD, GAD, and beyond. Neuropsychopharmacology, 42(1), 254–270. doi:10.1038/npp.2016.146. 27510423 PMC5143487

[ref25] Miller, A. H., & Raison, C. L. (2016). The role of inflammation in depression: From evolutionary imperative to modern treatment target. Nature Reviews Immunology, 16, 22–34. doi:10.1038/nri.2015.5. PMC5542678 26711676

[ref26] Munafò, M. R., Tilling, K., Taylor, A. E., Evans, D. M., & Davey Smith, G. (2017). Collider scope: When selection bias can substantially influence observed associations. International Journal of Epidemiology, 47(1), 226–235. doi:10.1093/ije/dyx206. PMC5837306 29040562

[ref27] Olena Yavorska, J. S. (2021). MendelianRandomization: Mendelian Randomization Package. R package version 0.5.1. Retrieved from https://CRAN.R-project.org/package=MendelianRandomization.

[ref28] Osimo, E. F., Baxter, L. J., Lewis, G., Jones, P. B., & Khandaker, G. M. (2019). Prevalence of low-grade inflammation in depression: A systematic review and meta-analysis of CRP levels. Psychological Medicine, 49(12), 1958–1970. doi:10.1017/s0033291719001454. 31258105 PMC6712955

[ref29] Perry, B. I., Upthegrove, R., Kappelmann, N., Jones, P. B., Burgess, S., & Khandaker, G. M. (2021). Associations of immunological proteins/traits with schizophrenia, major depression and bipolar disorder: A bi-directional two-sample Mendelian randomization study. Brain, Behavior, and Immunity, 97, 176–185. doi:10.1016/j.bbi.2021.07.009. 34280516 PMC7612947

[ref30] Pitsavos, C., Panagiotakos, D. B., Papageorgiou, C., Tsetsekou, E., Soldatos, C., & Stefanadis, C. (2006). Anxiety in relation to inflammation and coagulation markers, among healthy adults: The ATTICA study. Atherosclerosis, 185(2), 320–326. doi:10.1016/j.atherosclerosis.2005.06.001. 16005881

[ref31] R Core Team. (2021). R: A language and environment for statistical computing. (version 4.0.5 (2021-03-31) – ‘shake and throw’). Vienna, Austria: R Foundation for Statistical Computing. Retrieved from https://www.R-project.org/.

[ref32] Spitzer, R. L., Kroenke, K., Williams, J. B. W., & Löwe, B. (2006). A brief measure for assessing generalized anxiety disorder: The GAD-7. Archives of Internal Medicine, 166(10), 1092–1097. doi:10.1001/archinte.166.10.1092. 16717171

[ref33] Staley, J. R., Blackshaw, J., Kamat, M. A., Ellis, S., Surendran, P., Sun, B. B., … Butterworth, A. S. (2016). PhenoScanner: A database of human genotype-phenotype associations. Bioinformatics (Oxford, England), 32(20), 3207–3209. doi:10.1093/bioinformatics/btw373. 27318201 PMC5048068

[ref34] Swerdlow, D. I., Holmes, M. V., Kuchenbaecker, K. B., Engmann, J. E., Shah, T., Sofat, R., … Casas, J. P. (2012). The interleukin-6 receptor as a target for prevention of coronary heart disease: A Mendelian randomisation analysis. Lancet (London, England), 379(9822), 1214–1224. doi:10.1016/s0140-6736(12)60110-x. 22421340 PMC3316968

[ref35] Vogelzangs, N., Beekman, A. T., de Jonge, P., & Penninx, B. W. (2013). Anxiety disorders and inflammation in a large adult cohort. Translational Psychiatry, 3, e249. doi:10.1038/tp.2013.27. 23612048 PMC3641413

[ref36] Wensley, F., Gao, P., Burgess, S., Kaptoge, S., Di Angelantonio, E., Shah, T., … Danesh, J. (2011). Association between C reactive protein and coronary heart disease: Mendelian randomisation analysis based on individual participant data. British Medical Journal *(*Clinical Research Edition*)*, 342, d548. doi:10.1136/BMJ.D548. PMC3039696 21325005

[ref37] Ye, Z., Kappelmann, N., Moser, S., Davey Smith, G., Burgess, S., Jones, P. B., & Khandaker, G. M. (2021). Role of inflammation in depression and anxiety: Tests for disorder specificity, linearity and potential causality of association in the UK Biobank. EClinicalMedicine, 38, 100992. 10.1016/j.eclinm.2021.100992. 34505025 PMC8413248

