# Supplementary material for: The causal role of C-reactive protein and interleukin-6 on anxiety and depression symptoms and life satisfaction: Mendelian randomisation analyses in the HUNT study
Source: Psychol Med. 2023 May 23;53(16):7561–8. doi: 10.1017/S0033291723001290 (PMC10755231; doi:10.1017/S0033291723001290)
Supplement: Bekkevold et al. supplementary material [file S0033291723001290sup001.docx]

# Supplementary data

# The causal role of C-reactive protein and interleukin-6 on anxiety and depression symptoms and life satisfaction. Mendelian Randomization analyses in the HUNT Study.

1. The HUNT study and variables

The population-based HUNT Study has invited the adult population of the Nord-Trøndelag region, Norway to health surveys on four occasions: HUNT1 (1984-86), HUNT2 (1995-97), HUNT3 (2006-08) and HUNT4 (2017-19). The surveys include questionnaires, clinical examination and blood sampling, and a broad specter of phenotypes has been collected, as described in detail previously ([Krokstad et al., 2013](#_ENREF_15)). Approximately 70,000 participants of HUNT2 or HUNT3 have been genotyped. Methods of genotyping and imputation have been described elsewhere ([Brumpton et al., 2021](#_ENREF_3)). In HUNT2, serum CRP was measured in 4 out of 24 municipalities using a serum micro C-reactive protein ultra-sensitive assay methodology (Tina-quant(R), Roche, Basel, Switzerland) with measurement range of 0.1-300 mg/l ([HUNT Databank, 2022a](#_ENREF_11)). In HUNT3, CRP was measured in all participants using latex immunoassay methodology (Abbott, Clinical Chemistry, USA) with a measurement range of 0.1-160 mg/l ([HUNT Databank, 2022b](#_ENREF_12)). HADS score was collected in the baseline questionnaire in both HUNT2 and HUNT3. The HADS questionnaire consists of 14 questions (7 on depressive and 7 on anxiety symptoms), each with 4 ordered alternative answers (Suppl. table 1). The range of the total score is 0-42, and the sub-scores for anxiety symptoms (HADS-A) and depressive symptoms (HADS-D) both range from 0 to 21, where 21 represents the most severe symptoms. For participants with missing values for individual HADS items we calculated the total HADS-score, if ≥11 questions had valid values, by summarizing the available values, dividing by number of valid values and multiplying by 14. For the HADS-A and HADS-D subscales we similarly calculated the scores if ≥5 out of 7 answers were valid. We analyzed HADS and its subscales as continuous variables, and also analyzed elevated HADS-A and HADS-D scores as binary outcomes, where we defined HADS-A score of ≥8 as clinical anxiety, and HADS-D score of ≥8 as clinical depression ([Bjelland, Dahl, Haug, & Neckelmann, 2002](#_ENREF_1)). Life satisfaction was self-reported in the baseline questionnaire using the question: “Thinking about your life at the moment, would you say that you by and large are satisfied with life, or are you mostly dissatisfied?”, with seven ordered alternative answers ranging from 1: “very satisfied” to 7: “very dissatisfied”. We analyzed the life satisfaction score as a continuous variable. See Lysberg et al. ([Lysberg et al., 2021](#_ENREF_17)) for more information about the life satisfaction score.

| HADS-Anxiety | | | HADS-Depression | | |
| --- | --- | --- | --- | --- | --- |
| Statement | Answers | Points | Statement | Answer | Points |
| I feel tense or “wound up” | Most of the time | 3 | I still enjoy the things I used to enjoy | Definitely as much | 0 |
|  | A lot of the time | 2 |  | Not quite so much | 1 |
|  | From time to time, occasionally | 1 |  | Only a little | 2 |
|  | Not at all | 0 |  | Hardly at all | 3 |
| I get a sort of frightened feeling as if something awful is about to happen | Very definitely and quite badly | 3 | I can laugh and see the funny side of things | As much as I always could | 0 |
|  | Yes, but not too badly | 2 |  | Not quite as much now | 1 |
|  | A little, but it doesn´t worry me | 1 |  | Definitely not so much now | 2 |
|  | Not at all | 0 |  | Not at all | 3 |
| Worrying thoughts go through my mind | A great deal of the time | 3 | I feel cheerful | Not at all | 3 |
|  | A lot of the time | 2 |  | Not often | 2 |
|  | From time to time, but not too often | 1 |  | Sometimes | 1 |
|  | Only occasionally | 0 |  | Most of the time | 0 |
| I can sit at ease and feel relaxed | Definitely | 0 | I feel as if I am slowed down | Nearly all the time | 3 |
|  | Usually | 1 |  | Very often | 2 |
|  | Not often | 2 |  | Sometimes | 1 |
|  | Not at all | 3 |  | Not at all | 0 |
| I get a sort of frightened feeling like “butterflies” in the stomach | Not at all | 0 | I have lost interest in my appearance | Definitely | 3 |
|  | Occasionally | 1 |  | I don´t take as much care as I should | 2 |
|  | Quite often | 2 |  | I may not take quite as much care | 1 |
|  | Very often | 3 |  | I take just as much care as ever | 0 |
| I feel restless as I have to be on the move | Very much indeed | 3 | I look forward with enjoyment to things | As much as I ever did | 0 |
|  | Quite a lot | 2 |  | Rather less than I used to | 1 |
|  | Not very much | 1 |  | Definitely less than I used to | 2 |
|  | Not at all | 0 |  | Hardly at all | 3 |
| I get sudden feelings of panic | Very often indeed | 3 | I can enjoy a good book or radio or TV program | Often | 0 |
|  | Quite often | 2 |  | Sometimes | 1 |
|  | Not very often | 1 |  | Not often | 2 |
|  | Not at all | 0 |  | Very seldom | 3 |

Suppl. table 1: Items in Hospital Anxiety and Depression Scale questionnaire (English version)

1. Genetic instruments

| Instrument | SNP | Effect allele | Other allele | Source for curated instrument | Source for raw instrument |
| --- | --- | --- | --- | --- | --- |
| CRP-conservative and liberal | rs3093077 | C | A | Hartwig 2017 ([Hartwig, Borges, Horta, Bowden, & Davey Smith, 2017](#_ENREF_9)) | CCGC 2011([Wensley et al., 2011](#_ENREF_21)) |
| CRP-  conservative  and liberal | rs1205 | C | T |  |  |
| CRP-  conservative  and liberal | rs1130864 | A | G |  |  |
| CRP-  conservative  and liberal | rs1800947 | C | G |  |  |
| CRP-liberal | rs2794520 | C | T | Hartwig 2017 ([Hartwig, Borges, et al., 2017](#_ENREF_9)) | Dehghan 2011([Dehghan et al., 2011](#_ENREF_6)) |
| CRP-liberal | rs4420638 | A | G |  |  |
| CRP-liberal | rs1183910 | G | A |  |  |
| CRP-liberal | rs4420065 | C | T |  |  |
| CRP-liberal | rs4129267 | C | T |  |  |
| CRP-liberal | rs1260326 | T | C |  |  |
| CRP-liberal | rs12239046 | C | T |  |  |
| CRP-liberal | rs6734238 | G | A |  |  |
| CRP-liberal | rs9987289 | G | A |  |  |
| CRP-liberal | rs10745954 | A | G |  |  |
| CRP-liberal | rs1800961 | C | T |  |  |
| CRP-liberal | rs340029 | T | C |  |  |
| CRP-liberal | rs10521222 | C | T |  |  |
| CRP-liberal | rs12037222 | A | G |  |  |
| CRP-liberal | rs13233571 | C | T |  |  |
| CRP-liberal | rs2847281 | A | G |  |  |
| CRP-liberal | rs6901250 | A | G |  |  |
| CRP-liberal | rs4705952 | G | A |  |  |

Suppl. table 2: SNPs used in conservative and liberal CRP-instrument, and sources. CRP=C-reactive protein, SNP= single nucleotide polymorphism.

| Instrument | SNP | Beta | SE | Effect allele | Other allele | eaf | p | Units | Source for curated instrument | Source for raw instrument |
| --- | --- | --- | --- | --- | --- | --- | --- | --- | --- | --- |
| IL-6 | rs7529229 | 0,086 | 0,012 | C | T | 0,55 | 6,15E-12 | ln(pg/mL) | Ye et al 2021([Ye et al., 2021](#_ENREF_22)) | Swerdlow et al 2012([Swerdlow et al., 2012](#_ENREF_20)) |
| IL-6 | rs4845371 | 0,062 | 0,013 | C | T | 0,43 | 6,78E-07 | ln(pg/mL) |  |  |
| IL-6 | rs12740969 | 0,078 | 0,013 | G | T | 0,49 | 2,20E-09 | ln(pg/mL) |  |  |

Suppl. table 3: SNPs used in the two sample MR and association to IL-6. IL-6= Interleukin 6, eaf=effect allele frequency.

| Instrument | SNP | Effect allele | Other allele | Source for curated instrument | Source for raw instrument |
| --- | --- | --- | --- | --- | --- |
| CRP-sensitivity | rs112433451 | T | C | Ye et al 2021([Ye et al., 2021](#_ENREF_22)) | Ligthart et al 2018([Ligthart et al., 2018](#_ENREF_16)) |
| CRP-sensitivity | rs112689575 | A | G |  |  |
| CRP-sensitivity | rs115321806 | T | C |  |  |
| CRP-sensitivity | rs115585839 | T | G |  |  |
| CRP-sensitivity | rs116702045 | A | G |  |  |
| CRP-sensitivity | rs11811420 | C | G |  |  |
| CRP-sensitivity | rs1205 | T | C |  |  |
| CRP-sensitivity | rs1446975 | T | C |  |  |
| CRP-sensitivity | rs151313645 | A | C |  |  |
| CRP-sensitivity | rs17459069 | T | C |  |  |
| CRP-sensitivity | rs187173349 | A | T |  |  |
| CRP-sensitivity | rs28421518 | A | T |  |  |
| CRP-sensitivity | rs3093059 | A | G |  |  |
| CRP-sensitivity | rs35370436 | A | G |  |  |
| CRP-sensitivity | rs35485101 | A | T |  |  |
| CRP-sensitivity | rs3806186 | T | G |  |  |
| CRP-sensitivity | rs55688443 | T | G |  |  |
| CRP-sensitivity | rs61821567 | A | C |  |  |
| CRP-sensitivity | rs6686576 | A | G |  |  |
| CRP-sensitivity | rs72698563 | A | T |  |  |
| CRP-sensitivity | rs7517317 | T | C |  |  |
| CRP-sensitivity | rs7519020 | A | G |  |  |
| CRP-sensitivity | rs7521729 | A | C |  |  |
| CRP-sensitivity | rs77013776 | A | C |  |  |
| CRP-sensitivity | rs1205 | C | T | Ye et al 2021([Ye et al., 2021](#_ENREF_22)) | CCGC 2011([Wensley et al., 2011](#_ENREF_21)) |
| CRP-sensitivity | rs3093077 | C | A |  |  |
| CRP-sensitivity | rs1130864 | A | G |  |  |
| CRP-sensitivity | rs1800947 | C | A |  |  |

Suppl. table 4: SNPs used in CRP-sensitivity instrument and sources. CRP=C-reactive protein, SNP= single nucleotide polymorphism.

1. Method for assessment of MR assumptions and other sensitivity analyses

To test the relevance assumption, we estimated the F-statistics for the instruments, where a value below 10 was considered as suggesting a weak association of the SNP with the exposure. As the BOLT-LMM software does not calculate F-statistics for individual SNPs, we calculated the “pseudo” F-statistics using the following formula:

$$F=\frac{\beta_{x}^{2}}{{SE}_{x}^{2}}$$

To estimate an overall R squared and f-statistic for the CRP-instruments we created allele counts of CRP-increasing SNPs in the conservative and liberal CRP instruments respectively and ran linear regression with CRP as outcome and the allele counts as exposure variables. To evaluate the exclusion restriction and independence assumptions we used the R-package Phenoscanner ([Kamat et al., 2019](#_ENREF_13); [Staley et al., 2016](#_ENREF_19)) to search through the NHGRI-EBI GWAS-catalog ([Buniello et al., 2019](#_ENREF_4)) for associations (with p-value < 5x10^-8^) between the instrument SNPs and possible confounders or risk factors that could indicate pleiotropic pathways. Additionally, we performed logistic and linear regression analyses between the allele counts of CRP-increasing SNPs and likely confounders measured in HUNT. Another sign of pleiotropy is if there is heterogeneity in the MR estimate across the SNPs. We assessed this by the Cochran Q-statistic and its corresponding p-value with the null-hypothesis that the effect of each instrument variable is homogenous. Hence, a small p-value could reflect pleiotropy ([Greco, Minelli, Sheehan, & Thompson, 2015](#_ENREF_8)). To examine the exclusion restriction assumption, we applied a set of sensitivity analyses: With MR-Egger ([Burgess & Thompson, 2017](#_ENREF_5)) we allow the regression line for the estimated causal exposure-outcome effect to intercept the y axis at non-zero values for y. If the intercept of the y-axis is located far from zero, this could indicate directional horizontal pleiotropy. The coefficient of the MR-Egger line will reflect the causal effect of the exposure on the outcome if there is no measurement error and the pleiotropic effect is independent of the strength of the SNP-exposure effect (InSIDE assumption). Weighted median MR yields valid causal estimates if more than 50% of the SNP-exposure information comes from valid instrumental variable SNPs ([Bowden, Davey Smith, Haycock, & Burgess, 2016](#_ENREF_2)). Additionally, we performed weighted mode MR to avoid the influence of outliers ([Hartwig, Davey Smith, & Bowden, 2017](#_ENREF_10); [Sanderson et al., 2022](#_ENREF_18)). The MR-Egger was done with the “MendelianRandomization” package in R taking into account that some of the SNPs were in linkage disequilibrium. For the weighted median analysis, we excluded all SNPs in linkage disequilibrium with a R2> 0.001 (rs2794520, rs3093077, rs1130864 and rs1800947), keeping the SNP with lowest p-value.

Recently, several MR studies ([Georgakis et al., 2020](#_ENREF_7); [Kappelmann et al., 2021](#_ENREF_14); [Ye et al., 2021](#_ENREF_22)) have used a genetic instrument, containing 24 SNPs in the *CRP* region, based on a GWAS by Ligthart et al ([Ligthart et al., 2018](#_ENREF_16)). As a final sensitivity analysis we performed IVW MR using this instrument. We combined these SNPs with the SNPs from the conservative instrument and performed IVW MR taking correlatedness into account. One SNP (rs 1205) was present in both CRP conservative and the Ligthart instrument, another SNP (rs28421518) was not present in the LD-reference panel from the IEU GWAS database, hence we ended up with 26 SNPs in the new CRP instrument which we labeled “CRP-Sensitivity”.

1. Assessment of assumptions
2. Relevance assumption

| SNP | Effect allele | Other allele | MAF | Beta | SE | P-value | F-statistic | Sample N |
| --- | --- | --- | --- | --- | --- | --- | --- | --- |
| rs3093077 | C | A | 0.06 | 0.309 | 0.0211 | 1.5e-48 | 214.4 | 51348 |
| rs1205 | T | C | 0.34 | -0.267 | 0.0105 | 6.5e-142 | 643.3 | 51348 |
| rs1130864 | A | G | 0.31 | 0.212 | 0.0108 | 7.7e-86 | 385.5 | 51348 |
| rs1800947 | G | C | 0.07 | -0.362 | 0.0197 | 5.8e-75 | 335.6 | 51348 |

Suppl. table 5: F-statistics of SNPs in conservative CRP instrument used in one-sample MR (effect on log2(CRP levels)). SNP=single nucleotide polymorphism, MAF=minor allele frequency, SE=standard error.

| SNP | Effect allele | Other allele | MAF | Beta | SE | P-value | F-statistic | Sample N |
| --- | --- | --- | --- | --- | --- | --- | --- | --- |
| rs10745954 | G | A | 0.48 | -0.017 | 0.01 | 0.098 | 2.7 | 51348 |
| rs1183910 | A | G | 0.31 | -0.207 | 0.0108 | 2.1e-81 | 365.2 | 51348 |
| rs340029 | T | C | 0.36 | 0.055 | 0.0104 | 1.1e-07 | 28.2 | 51348 |
| rs10521222 | T | C | 0.04 | -0.171 | 0.0265 | 1.1e-10 | 41.6 | 51348 |
| rs2847281 | G | A | 0.41 | 0.044 | 0.0102 | 1.5e-05 | 18.8 | 51348 |
| rs4420638 | G | A | 0.21 | -0.299 | 0.0123 | 2.2e-131 | 594.8 | 51348 |
| rs4129267 | T | C | 0.38 | -0.133 | 0.0103 | 3.8e-38 | 166.8 | 51348 |
| rs2794520 | T | C | 0.34 | -0.266 | 0.0105 | 2e-141 | 641 | 51348 |
| rs3093077 | C | A | 0.06 | 0.309 | 0.0211 | 1.5e-48 | 214.4 | 51348 |
| rs1205 | T | C | 0.34 | -0.267 | 0.0105 | 6.5e-142 | 643.3 | 51348 |
| rs1130864 | A | G | 0.31 | 0.212 | 0.0108 | 7.7e-86 | 385.5 | 51348 |
| rs1800947 | G | C | 0.07 | -0.362 | 0.0197 | 5.8e-75 | 335.6 | 51348 |
| rs12239046 | C | T | 0.37 | 0.038 | 0.0104 | 0.00029 | 13.1 | 51348 |
| rs12037222 | A | G | 0.21 | 0.026 | 0.0123 | 0.032 | 4.6 | 51348 |
| rs4420065 | C | T | 0.4 | 0.149 | 0.0101 | 1.4e-48 | 214.6 | 51348 |
| rs1800961 | T | C | 0.05 | -0.138 | 0.0227 | 1.1e-09 | 37.1 | 51348 |
| rs6734238 | G | A | 0.41 | 0.066 | 0.0102 | 6.3e-11 | 42.7 | 51348 |
| rs1260326 | C | T | 0.33 | -0.103 | 0.0106 | 3.5e-22 | 93.8 | 51348 |
| rs4705952 | A | G | 0.25 | 0.015 | 0.0115 | 0.19 | 1.7 | 51348 |
| rs6901250 | A | G | 0.29 | -0.017 | 0.0111 | 0.11 | 2.5 | 51348 |
| rs13233571 | T | C | 0.13 | -0.073 | 0.015 | 1e-06 | 23.9 | 51348 |
| rs9987289 | G | A | 0.12 | 0.122 | 0.0153 | 1.5e-15 | 63.7 | 51348 |

Suppl. table 6: F-statistics of SNPs in liberal CRP instrument used in one-sample MR (effect on log2(CRP levels)). SNP=single nucleotide polymorphism, MAF=minor allele frequency, SE=standard error.

| SNP | Beta | SE | Effect allele | Other allele | EAF | P-value | F-statistic |
| --- | --- | --- | --- | --- | --- | --- | --- |
| rs7529229 | 0.086 | 0.012 | C | T | 0.55 | 6.15e-12 | 51.36 |
| rs4845371 | 0.062 | 0.013 | C | T | 0.43 | 6.78e-07 | 22.75 |
| rs12740969 | 0.078 | 0.013 | G | T | 0.49 | 2.2e-09 | 36 |

Suppl. table 7: F-statistics for SNPs used in two-sample MR(effect on IL-6(ln(pg/mL))). SNP=single nucleotide polymorphism, EAF=effect allele frequency, SE=Standard error.

| Instrument | R-squared | Adjusted R-squared | F-statistic |
| --- | --- | --- | --- |
| CRP-conservative (allele count) | 0.019 | 0.019 | 989.9 |
| CRP-liberal (allele count) | 0.038 | 0.038 | 2025.2 |

Suppl. table 8: R-squared from linear regression between count of CRP increasing alleles and log2 transformed CRP levels.

1. Exclusion restriction assumption and independence assumption

| SNP | Number of associations to non-CRP traits on Phenoscanner |
| --- | --- |
| rs1130864 | 0 |
| rs1205 | 0 |
| rs1800947 | 0 |
| rs3093077 | 0 |

Suppl. table 9: Results from query of SNPs in conservative CRP instrument for associations with non-CRP traits. Query performed March 30^th^ 2022. SNP=single nucleotide polymorphism, CRP=C-reactive protein

| SNP | Number of associations | Categories of traits |
| --- | --- | --- |
| rs2794520 | 3 | Inflammation, metabolic |
| rs4420638 | 102 | Ocular disease, dementia, BMI, CVD/CVD risk factors, lung function, life longevity, metabolic |
| rs1183910 | 12 | Cholelithiasis, CVD/CVD risk factors, metabolic |
| rs4420065 | 13 | Inflammation |
| rs4129267 | 15 | Allergy, asthma, inflammation, rheumatoid arthritis, eczema |
| rs1260326 | 166 | DM, liver, alcohol, CVD/CVD risk factors, Crohn’s disease, kidney function, gout, inflammation, hematology, BMI |
| rs12239046 | 10 | Inflammation |
| rs6734238 | 10 | Inflammation |
| rs9987289 | 31 | CVD/CVD risk factors, metabolic |
| rs1800961 | 23 | CVD/CVD risk factors, inflammation |
| rs12037222 | 20 | BMI,CVD/CVD risk factors |
| rs13233571 | 34 | Inflammation, CVD/CVD risk factors, gout |
| rs2847281 | 3 | Cancer, rheumatoid arthritis |
| rs4705952 | 6 | Inflammation |

Suppl. table 10: SNPs in the liberal CRP instrument that showed GWAS significant (p=5*10^-8^) associations to non-CRP traits and number of such associations. Query from Phenoscanner march 30^th^ 2022. We categorized the traits into broader groups in order to give an overview. SNP=single nucleotide polymorphism, BMI=body mass index, CVD=cardiovascular disease, DM=diabetes mellitus.

| SNP | Number of associations to other traits than IL-6 | Traits |
| --- | --- | --- |
| rs7529229 | 9 | Allergic diseases, c-reactive protein, coronary artery disease, hayfever, allergic rhinitis or eczema, monocyte percentage of white cells,  ”No blood clot, bronchitis, emphysema, asthma, rhinitis, eczema or allergy diagnosed by doctor”, rheumatoid arthritis, “Self-reported eczema or dermatitis” |
| rs4845371 | 1 | Coronary artery disease |
| rs12740969 | 0 |  |

Suppl. table 11: SNPs in the IL-6 instrument and GWAS significant (p=5*10^-8^) associations to traits other than IL-6 from Phenoscanner. Query from Phenoscanner march 30^th^ 2022. IL-6=interleukin-6.

| Instrument | Variable | Effect | CI 95% min | CI 95% max | P-value |
| --- | --- | --- | --- | --- | --- |
| CRP-conservative (allele count) | BMI | -0.015(units change) | -0.038 | 0.008 | 0.193 |
| CRP-conservative (allele count) | Smoking status | 0.997(OR) | 0.983 | 1.01 | 0.651 |
| CRP-conservative (allele count) | MI | 0.997(OR) | 0.97 | 1.026 | 0.859 |
| CRP-conservative (allele count) | Heart failure | 1.029(OR) | 0.969 | 1.093 | 0.353 |
| CRP-conservative (allele count) | RA | 1.002(OR) | 0.968 | 1.037 | 0.913 |

Suppl. table 12: The association between the CRP-conservative instrument (allele count of CRP increasing alleles) and possible confounders in HUNT, estimates per CRP-increasing allele. CRP=C-reactive protein, BMI=body mass index, MI=myocardial infarction, RA= rheumatoid arthritis, OR=odds ratio, CI 95% min= lower limit of 95% confidence interval, CI 95% max=upper limit of 95% confidence interval.

| Instrument | Variable | Effect | CI 95% min | CI 95% max | P-value |
| --- | --- | --- | --- | --- | --- |
| CRP-liberal (allele score) | BMI | -0.001(units change) | -0.011 | 0.009 | 0.814 |
| CRP-liberal (allele score) | Smoking status | 0.999(OR) | 0.994 | 1.005 | 0.839 |
| CRP-liberal (allele score) | MI | 0.988(OR) | 0.977 | 1.000 | 0.053 |
| CRP-liberal (allele score) | Heart failure | 1.002(OR) | 0.977 | 1.028 | 0.871 |
| CRP-liberal (allele score) | RA | 1.004(OR) | 0.989 | 1.019 | 0.605 |

Suppl. table 13: The association between the CRP-liberal instrument (allele count of CRP increasing alleles) and possible confounders in HUNT, estimates per CRP-increasing allele. CRP=C-reactive protein, BMI=body mass index, MI=myocardial infarction, RA= rheumatoid arthritis, OR=odds ratio, CI 95% min= lower limit of 95% confidence interval, CI 95% max=upper limit of confidence interval.

| Instrument | Variable | Effect | CI95% min | CI95% max | Pvalue |
| --- | --- | --- | --- | --- | --- |
| IL-6 (allele score) | BMI | 0.001 (units change) | -0.018 | 0.02 | 0.942 |
| IL-6 (allele score) | Smoking status | 1.008 (OR) | 0.997 | 1.019 | 0.169 |
| IL-6 (allele score) | MI | 1.002 (OR) | 0.979 | 1.026 | 0.844 |
| IL-6 (allele score) | Heart failure | 0.981 (OR) | 0.933 | 1.03 | 0.434 |
| IL-6 (allele score) | RA | 0.985 (OR) | 0.957 | 1.013 | 0.295 |

Suppl. table 14: The association between interleukin-6 allele count and possible confounders or pleiotropic factors, effect per IL-6 increasing allele. IL-6=interleukin-6, BMI=body mass index, MI=myocardial infarction, RA= rheumatoid arthritis, OR=odds ratio, CI 95% min= lower limit of 95% confidence interval, CI 95% max=upper limit of confidence interval.


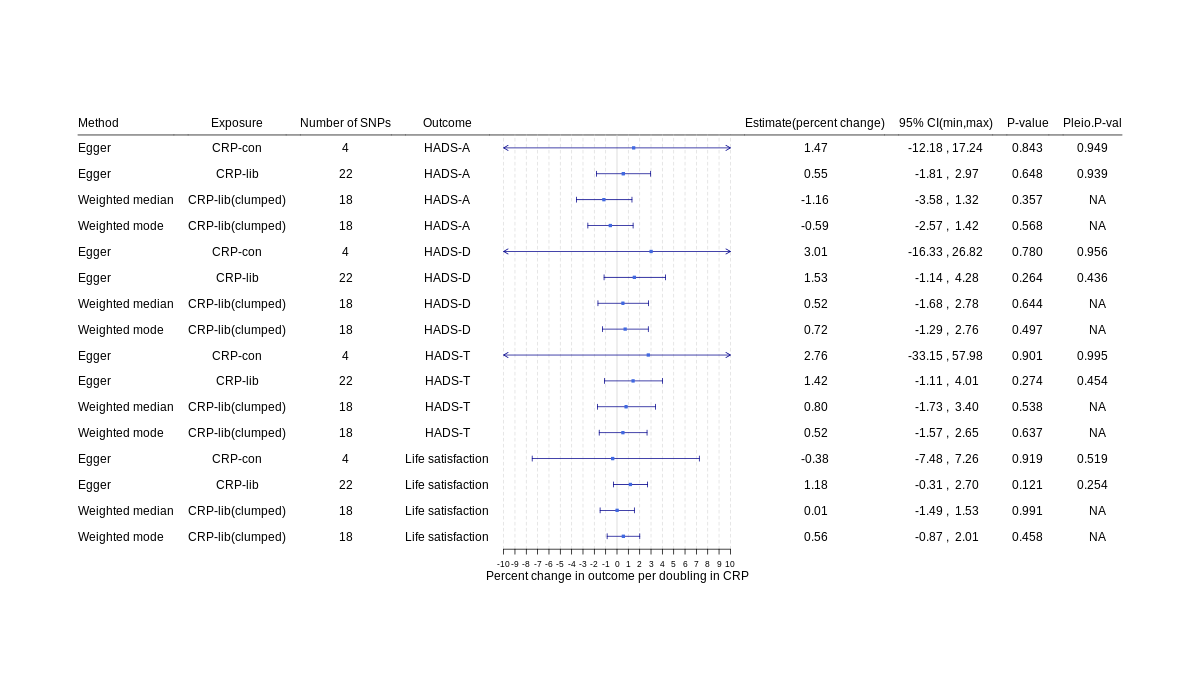


Suppl. figure 1: MR-Egger, weighted median MR and weighted mode MR for continuous outcomes. CRP=C-reactive protein, CRP-lib=CRP-liberal genetic instrument, CRP-con=CRP-conservative genetic instrument, SNP= single nucleotide polymorphism, HADS=hospital anxiety and depression scale, HADS-A=HADS-anxiety score, HADS-D=HADS-depression score, 95% CI= 95% confidence interval, Pleio.P-val=pleiotropy p-value.


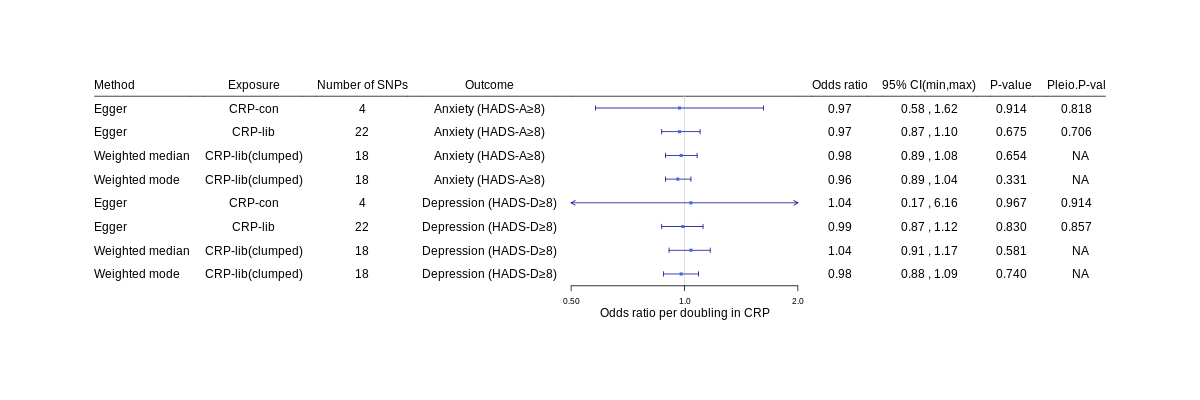


Suppl. figure 2: MR Egger, weighted median MR and weighted mode for binary outcomes. CRP=C-reactive protein, CRP-lib=CRP-liberal genetic instrument, CRP-con=CRP-conservative genetic instrument, SNP= single nucleotide polymorphism, HADS=hospital anxiety and depression scale, HADS-A=HADS-anxiety score, HADS-D=HADS-depression score, 95% CI= 95% confidence interval, Pleio.P-val=pleiotropy p-value.

1. Sensitivity analysis

| SNP | Effect allele | Other allele | Beta | SE | P-value | F-statistic |
| --- | --- | --- | --- | --- | --- | --- |
| rs115321806 | T | C | 0.161 | 0.04 | 6.9e-05 | 15.8 |
| rs7519020 | G | A | 0.024 | 0.012 | 0.04 | 4.2 |
| rs55688443 | T | G | -0.164 | 0.032 | 2.7e-07 | 26.4 |
| rs28421518* | A | T | -0.097 | 0.027 | 0.00035 | 12.8 |
| rs6686576 | A | G | 0.058 | 0.041 | 0.15 | 2.1 |
| rs187173349 | T | A | -0.192 | 0.019 | 6.3e-23 | 97.2 |
| rs112689575 | A | G | 0.036 | 0.042 | 0.38 | 0.8 |
| rs35485101 | A | T | 0.144 | 0.023 | 8e-10 | 37.8 |
| rs7517317 | T | C | 0.077 | 0.011 | 1.4e-11 | 45.7 |
| rs61821567 | A | C | 0.069 | 0.028 | 0.013 | 6.2 |
| rs1446975 | C | T | 0.073 | 0.01 | 1.5e-12 | 50.1 |
| rs35370436 | G | A | 0.161 | 0.022 | 4.5e-13 | 52.4 |
| rs72698563 | A | T | -0.165 | 0.029 | 1e-08 | 32.8 |
| rs17459069 | T | C | -0.172 | 0.025 | 3.3e-12 | 48.5 |
| rs77013776 | C | A | -0.321 | 0.024 | 3e-42 | 185.6 |
| rs151313645 | A | C | -0.205 | 0.041 | 4.4e-07 | 25.5 |
| rs3093077 | C | A | 0.309 | 0.021 | 1.5e-48 | 214.4 |
| rs1205 | T | C | -0.267 | 0.011 | 6.5e-142 | 643.3 |
| rs1130864 | A | G | 0.212 | 0.011 | 7.7e-86 | 385.5 |
| rs1800947 | G | C | -0.362 | 0.02 | 5.8e-75 | 335.6 |
| rs3093059 | G | A | 0.314 | 0.021 | 1.5e-49 | 219 |
| rs116702045 | A | G | -0.296 | 0.026 | 8.5e-30 | 128.5 |
| rs112433451 | C | T | -0.096 | 0.027 | 0.00033 | 12.9 |
| rs11811420 | C | G | 0.137 | 0.01 | 7.1e-40 | 174.7 |
| rs115585839 | T | G | 0.102 | 0.034 | 0.0028 | 9 |
| rs3806186 | G | T | -0.053 | 0.011 | 1.1e-06 | 23.8 |
| rs7521729 | A | C | 0.07 | 0.017 | 2.4e-05 | 17.8 |

Suppl. table 15: F-statistics for CRP-sensitivity instrument in the HUNT population (effect on log2(CRP levels)). *rs28421518 was removed from analysis as it was not present in the LD reference panel

| Exposure | R-squared | Adjusted R-squared | F-statistic |
| --- | --- | --- | --- |
| CRP-Sensitivity (allele count) | 0.014 | 0.014 | 722.5 |

Suppl. table 16: R-squared from linear regression between count of CRP increasing alleles in the CRP-sensitivity instrument and log2 transformed CRP levels.


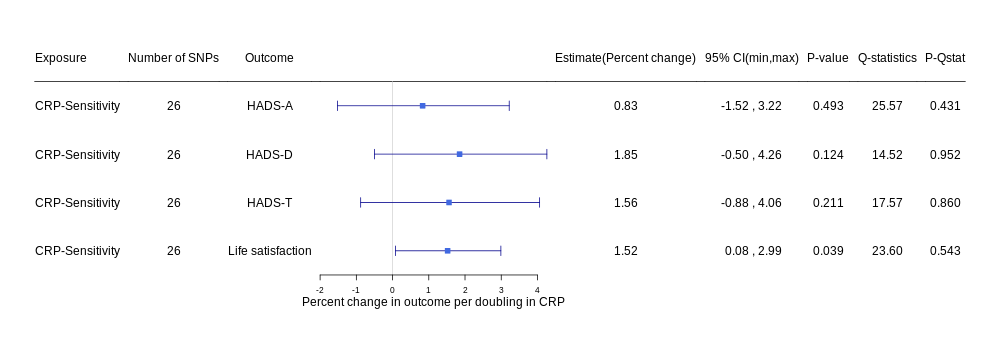


Suppl. figure 3: Results from one-sample MR for continuous outcomes with CRP-sensitivity instrument. CRP=C-reactive protein, SNP= single nucleotide polymorphism, HADS=hospital anxiety and depression scale, HADS-A=HADS-anxiety score, HADS-D=HADS-depression score, HADS-T= total HADS score, 95% CI= 95% confidence interval, P-Qstat= p-value for Q-statistic.


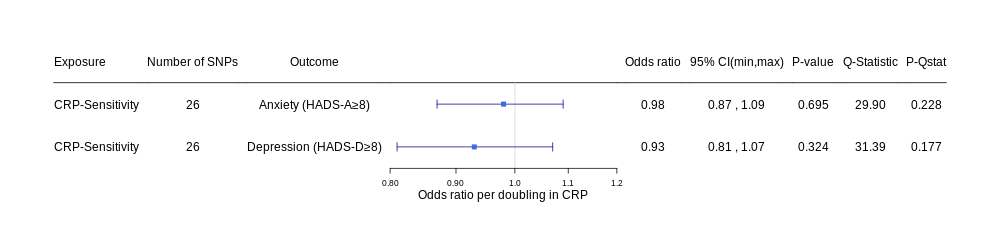


Suppl. figure 4: Results from one-sample MR for binary outcomes with CRP-sensitivity instrument. CRP=C-reactive protein, SNP= single nucleotide polymorphism, HADS=hospital anxiety and depression scale, HADS-A= HADS-anxiety score, HADS-D=HADS-depression score, HADS-T= total HADS score, 95% CI= 95% confidence interval, P-Qstat= p-value for Q-statistic.


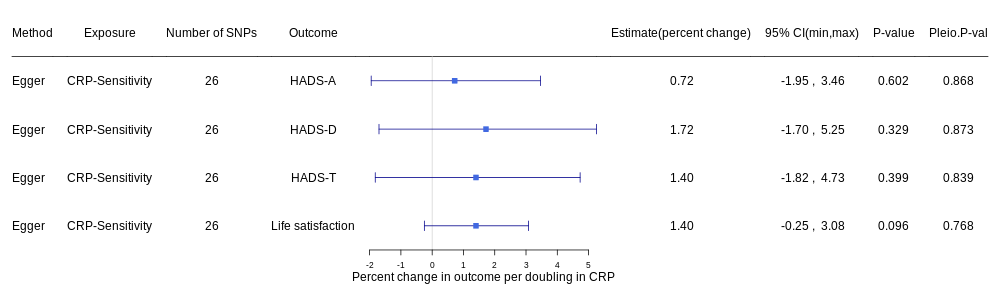


Suppl. figure 5: MR-Egger for continuous outcomes with CRP-sensitivity genetic instrument. CRP=C-reactive protein, CRP-lib=CRP-liberal genetic instrument, CRP-con=CRP-conservative genetic instrument, SNP= single nucleotide polymorphism, HADS=hospital anxiety and depression scale, HADS-A= HADS-anxiety score, HADS-D=HADS-depression score, 95% CI= 95% confidence interval, Pleio.P-val=pleiotropy p-value.


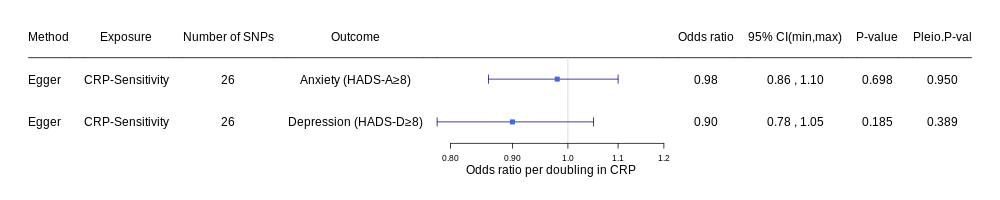


Suppl. figure 6: MR-Egger for binary outcomes with CRP-sensitivity genetic instrument. CRP=C-reactive protein, CRP-lib=CRP-liberal genetic instrument, CRP-con=CRP-conservative genetic instrument, SNP= single nucleotide polymorphism, HADS=hospital anxiety and depression scale, HADS-A= HADS-anxiety score, HADS-D=HADS-depression score, 95% CI= 95% confidence interval, Pleio.P-val=pleiotropy p-value.

- 1. References

Bjelland, I., Dahl, A. A., Haug, T. T., & Neckelmann, D. (2002). The validity of the Hospital Anxiety and Depression Scale: An updated literature review. *Journal of Psychosomatic Research, 52*, 69-77. doi:10.1016/S0022-3999(01)00296-3

Bowden, J., Davey Smith, G., Haycock, P. C., & Burgess, S. (2016). Consistent Estimation in Mendelian Randomization with Some Invalid Instruments Using a Weighted Median Estimator. *Genetic epidemiology, 40*(4), 304-314. doi:10.1002/gepi.21965

Brumpton, B. M., Graham, S., Surakka, I., Skogholt, A. H., Løset, M., Fritsche, L. G., . . . Willer, C. J. (2021). The HUNT Study: a population-based cohort for genetic research. *medRxiv*, 2021.2012.2023.21268305. doi:10.1101/2021.12.23.21268305

Buniello, A., MacArthur, J. A. L., Cerezo, M., Harris, L. W., Hayhurst, J., Malangone, C., . . . Parkinson, H. (2019). The NHGRI-EBI GWAS Catalog of published genome-wide association studies, targeted arrays and summary statistics 2019. *Nucleic Acids Research, 47*(D1), D1005-d1012. doi:10.1093/nar/gky1120

Burgess, S., & Thompson, S. G. (2017). Interpreting findings from Mendelian randomization using the MR-Egger method. *European Journal of Epidemiology, 32*(5), 377-389. doi:10.1007/s10654-017-0255-x

Dehghan, A., Dupuis, J., Barbalic, M., Bis, J. C., Eiriksdottir, G., Lu, C., . . . Chasman, D. I. (2011). Meta-analysis of genome-wide association studies in >80 000 subjects identifies multiple loci for C-reactive protein levels. *Circulation, 123*, 731-738. doi:10.1161/CIRCULATIONAHA.110.948570

Georgakis, M. K., Malik, R., Gill, D., Franceschini, N., Sudlow, C. L. M., Dichgans, M., . . . Slagboom, E. P. (2020). Interleukin-6 Signaling Effects on Ischemic Stroke and Other Cardiovascular Outcomes. *Circulation: Genomic and Precision Medicine, 13*(3), e002872. doi:doi:10.1161/CIRCGEN.119.002872

Greco, M. F., Minelli, C., Sheehan, N. A., & Thompson, J. R. (2015). Detecting pleiotropy in Mendelian randomisation studies with summary data and a continuous outcome. *Statistics in Medicine, 34*(21), 2926-2940. doi:10.1002/sim.6522

Hartwig, F. P., Borges, M. C., Horta, B. L., Bowden, J., & Davey Smith, G. (2017). Inflammatory Biomarkers and Risk of Schizophrenia. *JAMA Psychiatry, 74*, 1226. doi:10.1001/jamapsychiatry.2017.3191

Hartwig, F. P., Davey Smith, G., & Bowden, J. (2017). Robust inference in summary data Mendelian randomization via the zero modal pleiotropy assumption. *International Journal of Epidemiology, 46*(6), 1985-1998. doi:10.1093/ije/dyx102

HUNT Databank. (2022a). Variable description: Serum Micro C-reactive protein, HUNT 2. Retrieved from <https://hunt-db.medisin.ntnu.no/hunt-db/variable/7437>

HUNT Databank. (2022b). Variable description: Serum Micro C-reactive protein, HUNT 3. Retrieved from <https://hunt-db.medisin.ntnu.no/hunt-db/variable/158>

Kamat, M. A., Blackshaw, J. A., Young, R., Surendran, P., Burgess, S., Danesh, J., . . . Staley, J. R. (2019). PhenoScanner V2: an expanded tool for searching human genotype-phenotype associations. *Bioinformatics, 35*(22), 4851-4853. doi:10.1093/bioinformatics/btz469

Kappelmann, N., Arloth, J., Georgakis, M. K., Czamara, D., Rost, N., Ligthart, S., . . . Binder, E. B. (2021). Dissecting the Association Between Inflammation, Metabolic Dysregulation, and Specific Depressive Symptoms: A Genetic Correlation and 2-Sample Mendelian Randomization Study. *JAMA Psychiatry, 78*(2), 161-170. doi:10.1001/jamapsychiatry.2020.3436

Krokstad, S., Langhammer, A., Hveem, K., Holmen, T., Midthjell, K., Stene, T., . . . Holmen, J. (2013). Cohort Profile: The HUNT Study, Norway. *International Journal of Epidemiology, 42*, 968-977. doi:10.1093/ije/dys095

Ligthart, S., Vaez, A., Võsa, U., Stathopoulou, M. G., de Vries, P. S., Prins, B. P., . . . Alizadeh, B. Z. (2018). Genome Analyses of >200,000 Individuals Identify 58 Loci for Chronic Inflammation and Highlight Pathways that Link Inflammation and Complex Disorders. *American Journal of Human Genetics, 103*, 691-706. doi:10.1016/j.ajhg.2018.09.009

Lysberg, F., Bjerregaard Bertelsen, T., Lysberg, C., Høie, M., Espnes, G. A., & Innstrand, S. T. (2021). Change and stability: Within-person life satisfaction over a 20-year period using data from the HUNT survey. *Scandinavian journal of public health, 49*(8), 851-856. doi:10.1177/1403494820957439

Sanderson, E., Glymour, M. M., Holmes, M. V., Kang, H., Morrison, J., Munafò, M. R., . . . Davey Smith, G. (2022). Mendelian randomization. *Nature Reviews Methods Primers, 2*(1), 6. doi:10.1038/s43586-021-00092-5

Staley, J. R., Blackshaw, J., Kamat, M. A., Ellis, S., Surendran, P., Sun, B. B., . . . Butterworth, A. S. (2016). PhenoScanner: a database of human genotype-phenotype associations. *Bioinformatics, 32*(20), 3207-3209. doi:10.1093/bioinformatics/btw373

Swerdlow, D. I., Holmes, M. V., Kuchenbaecker, K. B., Engmann, J. E., Shah, T., Sofat, R., . . . Casas, J. P. (2012). The interleukin-6 receptor as a target for prevention of coronary heart disease: a mendelian randomisation analysis. *Lancet, 379*(9822), 1214-1224. doi:10.1016/s0140-6736(12)60110-x

Wensley, F., Gao, P., Burgess, S., Kaptoge, S., Di Angelantonio, E., Shah, T., . . . Danesh, J. (2011). Association between C reactive protein and coronary heart disease: mendelian randomisation analysis based on individual participant data. *British Medical Journal (Clinical research edition), 342*, d548. doi:10.1136/BMJ.D548

Ye, Z., Kappelmann, N., Moser, S., Davey Smith, G., Burgess, S., Jones, P. B., & Khandaker, G. M. (2021). Role of inflammation in depression and anxiety: Tests for disorder specificity, linearity and potential causality of association in the UK Biobank. *EClinicalMedicine, 38*, 100992. doi:<https://doi.org/10.1016/j.eclinm.2021.100992>
